# Evaluating the Hepatoprotective Effect of *Phyllanthus emblica* and *Panax ginseng* Powders on Acetaminophen‐Induced Liver Fibrosis in a Rat Model

**DOI:** 10.1002/fsn3.72135

**Published:** 2026-07-19

**Authors:** Fiza Javed, Nourah Harbi Almutairi, Wardha Tariq, Mah Noor, Shiza Javed, Usman Haider, Ghalia Shamlan, Gholamreza Abdi, Rana Muhammad Aadil

**Affiliations:** ^1^ National Institute of Food Science and Technology University of Agriculture Faisalabad Pakistan; ^2^ Department of Home Economics, College of Basic Education Public Authority for Applied Education and Training Kuwait Kuwait; ^3^ Department of Institute of Physiology and Pharmacology University of Agriculture Faisalabad Pakistan; ^4^ Department of Food Science and Nutrition, College of Food and Agriculture Sciences King Saud University Riyadh Saudi Arabia; ^5^ Department of Biotechnology, Persian Gulf Research Institute Persian Gulf University Bushehr Iran

**Keywords:** acetaminophen, combined effect, ellagic acid, ginsenosides, hepatoprotective, liver fibrosis, *Panax ginseng*, *Phyllanthus emblica* L.

## Abstract

Liver fibrosis is a progressive disorder, that originates from prolonged and persistent acetaminophen (APAP) exposure, a commonly used antipyretic and analgesic drug. If left untreated, fibrosis may lead to cirrhosis or hepatocellular carcinoma. *Phyllanthus emblica* L. and *Panax ginseng* C.A. Meyer possess hepatoprotective, antioxidant, and immunomodulatory properties. The aim of this study is to evaluate the individual and combined effects of *P. emblica* fruit and *P. ginseng* root powders against APAP induced liver fibrosis in a rat model. A 38‐day study trial was conducted using 20 male Wistar rats, which were divided into five groups, each group containing four rats. Hepatotoxic doses of APAP were administered for 10 days to induce early‐stage liver fibrosis in rats. A negative control group (G0) on normal diet without liver fibrosis, a positive control group with liver fibrosis on a normal diet (G1), and treatment groups (G2, G3, G4) receiving varying doses of *P. emblica* and *P. ginseng* were evaluated for liver function tests (alanine aminotransferase, aspartate aminotransferase, alkaline phosphatase, gamma glutamyl transferase, total bilirubin, albumin), oxidative stress biomarkers (glutathione), hematological indices and histopathological analysis. Results demonstrated that combination treatment G4 significantly improved liver function biomarkers, and normalized hematological parameters. However, *P. emblica* restored glutathione levels most effectively among all treatments. Histological examination revealed a marked reduction in necrosis and fibrosis. This study concluded that combined activity of bioactive components of *P. emblica* (vitamin C, ellagic acid, gallic acid) and *P. ginseng* (ginsenosides) significantly attenuated APAP‐induced hepatic damage and fibrosis, underscoring their potential role as complementary therapeutic options for drug‐induced liver disorders and halting fibrotic advancement.

## Graphical abstract

Schematic illustration of the experimental design and the hepatoprotective effect of *Phyllanthus emblica* and *Panax ginseng* against acetaminophen (APAP)‐induced fibrosis in Wistar rats. APAP administration leads to NAPQI formation and accumulation, which causes GSH depletion and HSC activation. This ultimately becomes the primary cause of liver fibrosis. Intervention of botanical powders of *P. emblica* and *P. ginseng* individually and collectively led to significant improvements. Liver function biomarkers such as ALT, AST, ALP, and GGT improved. Moreover, albumin and bilirubin levels were also recovered. Liver histopathology showed improvements in liver tissue structure such as minimum steatosis, inflammation, and hepatic cells with clear nuclei. Moreover, hematological parameters were also restored.

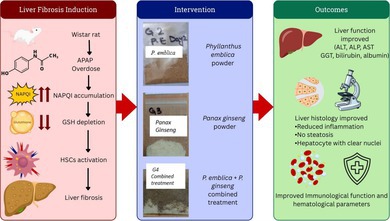

**Abbreviations**
ALP: alkaline phosphatase
ALT: Alanine aminotransferase
APAP: acetaminophen
AST: aspartate aminotransferase
GGT: gamma glutamyl transferase
GSH: glutathione
HSCs: hepatic stellate cells
NAPQI: N‐acetyl‐parabenzoquinone imine

## 1 Introduction

Liver being a central organ of human body comes across an array of circulatory elements including external agents and metabolites such as alcohol, drugs as well as pathogens. Prolonged exposure to these substances may lead to liver injury. Liver fibrosis is a response to chronic liver injury. All hepatocellular injuries stimulate a natural wound healing process known as “Fibrogenesis.” The activation of this pathway causes the space of Disse to receive fibrogenic components such as collagen and fibrillar proteins (e.g., elastin) of extracellular matrix (ECM), which serves as a purpose to isolate the damaged part of the liver for its repair. The primary cells in the wounded liver that produce ECM are hepatic stellate cells (HSCs). HSCs are the primary locations for vitamin A storage in normal liver and are found in space of Disse. After persistent injury, HSCs activate or transform themselves into cells that resemble myofibroblast gaining fibrogenic, contractile and pro‐inflammatory characteristics. When HSCs are activated, two main processes take place that encourage these cells' fibrogenic response. First HSCs become directly fibrogenic by altering their gene expression pattern, which leads to an increase in ECM synthesis and deposition. Second, after cellular activation HSCs proliferation rates rise. Liver fibrosis is one of the many chronic illnesses linked to ECM dysregulation. Acetaminophen (N‐acetyl‐p‐aminophenol [APAP]) overdose induces liver fibrosis by yielding a reactive metabolite N‐acetyl‐para‐benzoquinone imine (NAPQI) and depleting glutathione (GSH), a vital antioxidant that detoxifies NAPQI. Due to quick depletion of GSH stores, NAPQI attaches to proteins of cells and the mitochondria, creating adducts that interfere with mitochondrial respiration and increase oxidative stress. NAPQI accumulation leads to the development of hepatic necrosis. HSCs are activated by reactive oxygen species (ROS), which promote ECM deposition that increases up to 6–8 folds and collagen type I and II accumulation. Chronic activation of fibrogenic pathway leads to reduced breakdown of type 1 collagen which ultimately results in type 1 collagen accumulation in ECM that is considered as a characteristic feature of fibrosis (Acharya et al. 2021; Akkız et al. 2024; Alwahsh et al. 2019; Bataller and Brenner 2005; Einafshar et al. 2025; Friedman 2003; Gabele et al. 2003; Kisseleva and Brenner 2021; Li et al. 2023; Licata et al. 2022; Luangmonkong et al. 2023; Mitchell, Jollow, Potter, Davis, et al. 1973; Ramos‐Tovar and Muriel 2020). Although liver fibrosis was originally thought to be permanent, a large number of experiments and clinical researches have shown that fibrosis may be reversed if the underlying cause has been eliminated. HSC inactivation, ECM breakdown, and fibrotic tissue remodeling are linked to fibrosis regression (Bataller and Brenner 2005). These findings encouraged the exploration of novel antifibrotic agents capable of limiting fibrosis, Plants are rich in bioactive compounds that can offer hepatoprotective, antioxidant, anti‐inflammatory, antibacterial, immunomodulatory, and antiviral activities (Elzaiat et al. 2024; Bui et al. 2025; Khawaja et al. 2025; Wasim Sajid et al. 2025; Ullah et al. 2025; Usman et al. 2025; Babou et al. 2026). This makes them vital to alleviate APAP‐induced hepatic injury and widely relied upon by 80% of the global population (Akhtar et al. 2023). *Phyllanthus emblica* L. commonly known as amla or Indian gooseberry belongs to the family of Euphorbiaceae. It is abundant in vitamin C, tannins, phenolic compounds, flavonoids, carbohydrates and amino acids. *P. emblica* fruit significantly contains ellagic acid, gallic acid, emblicanin‐a, emblicanin‐b, chebulinic acid, quercetin, chebulagic acid, ascorbic acid, etc., which contribute to its hepatoprotective and antioxidant potential (Talreja et al. 2021). Antioxidants have been reported to inhibit ECM deposition caused by chemicals (e.g., thioacetamide) and surgical induction (e.g., bile duct ligation) (Casas‐Grajales et al. 2019). Previous studies on *P. emblica* have mainly focused on leaves or extract based systems. However comparative evidence suggests that fruit fractions demonstrate enhanced antioxidant potential than leaves, with lower IC_50_ values (3.20 vs. 6.10 μg/mL) indicated higher free radical scavenging activity and higher phenolic content (Orabi et al. 2023). Higher concentrations of gallic acid and vitamin C (282 mg/100 g of fresh fruit) in fruit pulp, indicates its strong antioxidant and antifibrotic properties when compared to leaves (19 mg/100 g fresh fruit) (Paik et al. 2020). Therefore, the use of pulp in present study provides a physiologically relevant and underexplored model for liver fibrosis.

Previous research shows that *P. emblica* is used in the treatment of liver cirrhosis, liver damage from alcohol and hepatitis (Chaudhary et al. 2024). Carbon tetrachloride (CCl_4_) is a toxic substance for the liver that leads to hepatic necrosis, pro‐inflammatory and pro‐fibrotic cytokine release which eventually leads to liver fibrosis and cirrhosis after prolonged exposure (Zhang et al. 2023). *P. emblica* extract is proven to reverse the hepatotoxicity by reducing serum peroxide, alkaline phosphatase (ALP), and glutamate‐pyruvate transaminase (GPT) caused by the chronic and acute CCl_4_ administration (Ikram et al. 2021).

*Panax ginseng* C.A. Meyer belongs to the Araliaceae family and ginsenosides found in *P. ginseng* are primary bioactive compounds, which possess diverse therapeutic properties, such as antihypertensive, antioxidant, anti‐inflammatory, anti‐fibrotic, antitumor and hypoglycemic actions. This broad pharmacological profile has made *P. ginseng* a cornerstone of East Asian traditional medicine for over two thousand years. Alanine aminotransferase (ALT) and aspartate aminotransferase (AST) levels are one of the main indicators that are screened to check effectiveness of ginseng against hepatocyte damage (Kim et al. 2021; Liu et al. 2021; Na et al. 2021). The bioactive compounds of *P. ginseng* include ginsenosides (Rb1, Rb2, Rd, Rg1, Rg3), polysaccharides, peptides, and fatty acids, which are obtained from different parts of ginseng that is, seed, root, stem, leaves, flowers, and fruits. In China, ginseng is known as “king of all herbs” and contains “Ginsenoside Rh1” that possesses hepatoprotective properties, prevents and manages liver cancer, exerts anti‐fibrotic, anti‐hyperlipidemic and hepatoprotective effects in nonalcoholic fatty liver diseases (NAFLD) (Fan et al. 2020; Kang et al. 2021; Lu et al. 2022). The study aims to investigate the hepatoprotective effect of combined treatment of *P. emblica* and *P. ginseng* and to evaluate whether their combined activity can attenuate and halt the progression of APAP‐induced liver fibrosis towards advanced stages such as cirrhosis.

## 2 Materials and Methods

### 2.1 Procurement of Raw Material

*P. emblica* fruits and *P. ginseng* roots were procured from well‐established and reputable vendors in the local market of Faisalabad. This approach ensured that authentic and high‐quality material was obtained, thus leading to accurate and valid experimental outcomes. Chemicals required for research analysis were obtained from a scientific store.

### 2.2 Preparation of Raw Materials

*P. emblica* fruits and *P. ginseng* roots were washed and dried. Raw material was dried by hot air oven drying method at a temperature of 55°C for 12 h, ground in an electric grinder and stored (Rathour et al. 2022).

### 2.3 Proximate Analyses of Raw Materials

*P. emblica* fruits and *P. ginseng* roots powders were analyzed. Proximate analyses which include moisture, crude fat, crude fiber, crude protein, and ash were performed according to the method suggested in literature (AACC 2016). The analyses of moisture (using hot air oven method), crude fat (using a Soxhlet apparatus), crude protein (using the Kjeldahl method; digestion, distillation, and titration), ash using muffle furnace (MF1/02, PCSIR, Pakistan), and crude fiber (using the Labconco Fibertech system, Labconco Corporation, Kansas, USA).

### 2.4 *P. emblica* and *P. ginseng* Extracts Preparation for Antioxidant Assay

#### 2.4.1 Preparation of *P. emblica* Extract

For preparation of aqueous extract of *P. emblica* , 120 g of amla powder was dissolved in 600 mL of distilled water. This mixture was then heated in a water bath for 20 min at a temperature of 60°C. After cooling to room temperature, the mixture was filtered through Whatman No. 1 filter paper to remove solid particles. Sediments were discarded whereas the solution underwent centrifugation at 6000 rpm for 15 min and filtered to remove fine residues. The next step involved storage of the filtrate at 4°C (Bin Fazal and Ahmad 2024; Burnice et al. 2024).

#### 2.4.2 Preparation of *P. ginseng* Extract

Ginseng sample was identified and authenticated at the Department of Botany, University of Agriculture, Faisalabad, Pakistan. Methanol extraction was performed using the maceration method at a ratio of 1:6 (1 g plant material in 6 mL methanol). Samples were incubated in methanol for 3 days at room temperature with occasional shaking. After three successive methanolic extraction cycles, the resulting viscous filtrates were subjected to water bath drying. The extraction was repeated three times with a four‐day interval between each cycle. The extracts were stored in −4°C and mixed in distilled water (Hussain et al. 2021).

The extracts were prepared for antioxidant analyses; detailed phytochemical profiling was beyond the scope of the present study.

### 2.5 Antioxidant Assay

2,2‐diphenyl‐1‐picrylhydrazyl (DPPH) analysis is the most common way to apprehend the antioxidant ability of a substance. The process involved dissolving the sample in 0.12 mL of DPPH solution in a test tube. The ratio was kept at 4:1 mL, respectively. The solution was then placed in a dark place and absorbance was checked at 520 nm by a UV/Visible Spectrophotometer (YH5300, Hitachi, Japan), with control and blank samples. The following formula was applied to check antioxidant activity (Naeem et al. 2022).

$$\text{Reduction of absorbance}=\frac{\mathrm{AB}-\mathrm{AA}}{\mathrm{AB}}\times 100$$

AB, absorbance of blank sample; AA, absorbance of tested extract sample.

### 2.6 Experimental Design

This study is purely a randomized controlled animal experiment. A total of 20 male Wistar strain albino rats weighing between 180–200 g, aged 8–12 weeks were procured and housed in well‐ventilated metal cages inside the animal room located in the University of Agriculture, Faisalabad, under the National Institute of Food Science and Technology, with the consent of the Institutional Biosafety and Bioethics Committee (D#2272/ORIC). The study duration was 38 days, which included an acclimatization period of 7 days and an experimental period of 31 days which consisted of *P. emblica* and *P. ginseng* treatment administration on an APAP induced liver fibrosis rat model. The rats were then randomly classified into five groups, and each group contained four rats (*n* = 4) (Nasir et al. 2025). However, for a liver fibrosis study, the relatively small sample size represents a limitation and may affect the statistical power of the analysis. Animals were monitored for general health and welfare throughout the study. Healthy rats that exhibited normal feeding and behavioral patterns, and a weight range of 180–200 g after the acclimatization period were selected for the experiment. Whereas animals that showed any signs of illness, abnormal behaviors and weight variations were excluded from the study. G0 was the negative control group, G1 was the positive control group, and the other three groups G2, G3, and G4 were treated with various concentrations of *P. emblica* and *P. ginseng* to investigate their individual and combined effects, as shown in Table 1. Due to practical constraints, blinding was not feasible in the treatment phase, but during outcome assessment blinding was applied where the investigator and pathologist were unaware of group allocation. The effectiveness of both herbal plants was analyzed in each treatment group and blood samples were collected at the end of the experiment. The following parameters were assessed: biomarkers of liver function including liver enzymes, bilirubin profile, albumin, GSH levels, hematological parameters, and liver histopathology. The weight was also monitored at various points throughout the study.

**TABLE 1:** Experimental groups and dietary treatments.

Group	Condition	Diet	Duration (days)
G0	No disease	Normal diet	31
G1	Liver fibrosis	Normal diet	31
G2	Liver fibrosis	2.5 g/kg of body weight *P. emblica*	31
G3	Liver fibrosis	500 mg/kg of body weight *P. ginseng*	31
G4	Liver fibrosis	2.5 g/kg of body weight *P. emblica* and 500 mg/kg of body weight *P. ginseng*	31

#### 2.6.1 Dose Selection

The doses of *P. emblica* and *P. ginseng* were selected based on previously reported efficacy studies and safety data. Acute toxicity studies demonstrate that *P. emblica* has a favorable safety profile, with no adverse effects observed in extract doses ranging from 2000 to 5000 mg/kg and a lethal dose (LD50) exceeding 5000 mg/kg in rats, and had no antagonistic effects on the liver, kidney, and heart (Anto et al. 2022). Efficacy studies show that *P. emblica* has hepatoprotective activity at 500 mg/kg in high‐fat diet‐induced NAFLD, enhanced activity of antioxidant enzymes at a dose of 200 mg/kg, and showed maximum anti‐inflammatory activity at 700 mg/kg (Golechha et al. 2014; Huang et al. 2017; Uddin et al. 2016). Based on the findings and the safety profile of *P. emblica* , a dose of 2.5 g/kg body weight was selected for the present study.

Similarly, *P. ginseng* has a wide safe range, an oral LD50 greater than 5000 mg/kg and no adverse effects when given to rats for 20 days at doses of 4000 mg/kg (Carabin et al. 2000). Another study where ginseng extract was administered and no adverse effects were reported at doses up to 2000 mg/kg (Park et al. 2013). Previous studies have reported hepatoprotective effects of ginseng within a range of 100–500 mg/kg dose range, supporting the selection of 500 mg/kg as an effective dose.

While previous studies have predominantly evaluated extracts of both plants, the present study explores their efficacy in powdered form. Since powders contain lower concentrations of individual bioactive constituents than extracts, relatively high doses are commonly required to achieve biologically relevant exposure. The effective dose range documented in the literature served as a guide for the doses chosen, which were deemed suitable for assessing the hepatoprotective activity of *P. emblica* and *P. ginseng* .

### 2.7 Induction of Liver Fibrosis

Hepatic fibrosis was induced by administration of APAP 1 g/kg of body weight per day orally for 10 days (Aly et al. 2020). Commercially available APAP tablets (500 mg) were obtained. The required number of tablets were finely crushed into a powder and dissolved in distilled water to prepare a uniform suspension of known concentration. The suspension was freshly prepared prior to each administration to ensure dose and stability. The rats weighed around 180–200 g. So, the dosage of APAP per rat was adjusted according to body weight which was around 200 mg per rat. The suspension was administrated orally via gastric gavage. The final administration volume was 2 mL per rat, which ensured safe administration of dosage for each rat.

The APAP dose and treatment duration were selected based on previously validated experimental studies investigating APAP‐induced hepatic fibrosis. The study reported significant hepatic injury accompanied by histopathological evidence of portal fibrosis and alterations in profibrotic mediators, including TNF‐α and TNF‐β (Aly et al. 2020). Furthermore, APAP‐induced liver injury is shown to progress from an early necro‐inflammatory phase to reparative fibrosis within a short duration of one to five days. APAP was given to fasting rats at a single dose of 1000 mg/kg bodyweight. It was reported that after two days of APAP administration necrosis and inflammation were developed. whereas, reparative fibrosis with elevated liver function enzymes were evident by day five (Tsuji et al. 2020). In addition, a study where liver response was investigated after second dose of APAP. The purpose was to assess whether a second dose of APAP changes the liver response from acute liver injury and necrosis to fibrogenesis (early fibrosis development). Mice were exposed to toxic doses of APAP, which were administered with an interval of 3 days. After the second dose of APAP increased ECM deposition, tissue remodeling, inflammation and abnormal nuclear changes such as accumulation of irregular small shaped nuclei and early fibrogenic changes were observed (Alwahsh et al. 2019). Collectively these studies indicate that the selected APAP dose and treatment duration are sufficient to induce fibrosis‐associated hepatic alteration and therefore support the present experimental protocol.

### 2.8 Collection of Samples

At the end of the experiment, rats were subjected to short‐term chloroform inhalation anesthesia. To ensure scientific reliability, a controlled anesthesia dose of 0.05 mL/L of container volume was used. Chloroform was applied to a sterile cotton and placed in a glass desiccator. A perforated platform blocked the direct contact of chloroform and rats. Rats were placed in the desiccator for a maximum of 30 s, until they appeared drowsy and reduced voluntary movement was observed. Rats were immediately removed from the glass desiccator (Aledani et al. 2020; Farooq et al. 2022; Rahmani et al. 2024). However, the use of chloroform may not present as the most standardized approach due to its toxicity and animal welfare concerns, compared to agents such as Isoflurane or Ketamine‐xylazine. Future studies are encouraged to adopt these standard anesthetic protocols.

The neck area was shaved to make a clear cut with a sterile scalpel blade on the jugular vein of rats. After this, a blood sample was collected into a labeled vial. The blood samples for serum biochemical analyses were collected in yellow top head vials. Blood samples for serum analyses were centrifuged at 700 rpm for 15 min after which the serum was separated from the clotted blood sample into plain tubes with the help of a pipette. Serum was stored at 4°C until the day of analysis. Whereas, the samples for complete blood count tests were collected in purple top head vials and after transferring blood to the vial, it was gently mixed so that the anti‐coagulants mix well to prevent blood from clotting (Chukwudoruo and Iheme 2021).

### 2.9 Determination of Liver Serum Biomarkers

Liver enzyme biomarkers were measured which included AST, ALT, ALP, gamma glutamyl transferase (GGT), total bilirubin, and albumin protein detected in the serum of rats according to the available kits and protocol prescribed by the manufacturer (Bagchi et al. 2025).

### 2.10 Determination of Oxidative Stress Markers

Liver tissues of the rats were homogenized with 0.1 M tris(hydroxymethyl)aminomethane hydrochloride (Tris HCl) buffer (pH 7.4) and centrifugation were done afterwards at 4°C, 2000 rpm for 10 min. A clear supernatant liquid was obtained to determine the results of oxidative stress biomarkers (Bagchi et al. 2025). GSH depletion is the earliest and primary event in APAP‐induced hepatotoxicity, as it directly leads to NAPQI accumulation (Chidiac et al. 2023). Since hepatocellular injury and downstream oxidative stress are a consequence of GSH depletion, GSH was selected as a primary biomarker in the present study. GSH content was measured according to the method described by (Islam et al. 2021). 10% of trichloroacetic acid was added to the liver tissue sample and then centrifuged so that the proteins could be separated. 0.1 mL clear liquid that was obtained and mixed with 2 mL of phosphate buffer which had a pH of 8.4, 0.5 mL of 5,5′‐dithiobis (2‐nitrobenzoic acid), and 0.4 mL of distilled water. Absorbance was measured at 412 nm using UV–VIS spectrometer (Shimadzu UV PC‐1600, Japan). The content of GSH was than written as micromole per gram (μmol/g) of tissue.

### 2.11 Histopathology of Liver

For liver histopathology, rat liver tissue was submerged in 10% phosphate buffered formalin. Tissue was then placed in a tissue processor machine and inserted in paraffin to get liver tissue histological specimen. Afterwards for biopsy evaluation 5 μm cuts were made and all parts were stained by hematoxylin and eosin stain (H&E) for histopathological evaluation and were observed under a microscope (Hamad Shareef et al. 2022). Due to limited laboratory resources, fibrosis‐specific staining was not performed in the current study. Therefore, the fibrosis‐related findings were interpreted based on H&E‐associated histological alterations. Future studies should include collagen‐specific staining techniques for definitive validation of hepatic fibrosis.

### 2.12 Statistical Analysis

All experimental groups consisted of four individual rats (*n* = 4 biological replicates per group). A single biochemical measurement was obtained from each animal, and the four resulting values per group were used to calculate the final data. Results were expressed as mean ± standard deviation (SD). All pairwise comparison tests were applied to check homogeneity among groups. Statistical differences among treatments were evaluated using completely randomized design (CRD). Data was analyzed using one‐way ANOVA, followed by Tukey's honestly significant difference (HSD) post hoc test. A *p* < 0.05 value was considered significant. Data analysis was performed using Statistix software version 8.0 (Montgomery 2019).

## 3 Results and Discussion

### 3.1 Proximate Composition of *P. emblica* and *P. ginseng*

Table 2 presents the proximate composition and antioxidant profiles of *P. emblica* and *P. ginseng* . Proximate analyses of *P. emblica* recorded an ash content which was consistent with values (2.1%–2.45%) reported in previous research. The protein content was similar to earlier findings of 3.5%, and the carbohydrate content remained near the range of 84.4%–86.5%, confirming carbohydrates as the predominant constituents of the fruit. A minimal fat concentration was observed, aligning with the low lipid nature of amla (Sonkar et al. 2020). Fiber levels reached slightly lower than 12% reported by existing literature (Tewari et al. 2019). It was probably due to different sample forms used for research. Moisture content of *P. emblica* , which differs from the previously mentioned research reference (84.2% ± 86.9%), confirming its typically high‐water composition. The prominent difference is due to the different forms of samples, since this research has used dried powdered form of amla.

**TABLE 2:** Proximate/antioxidant analyses of *P. emblica* and *P. ginseng* .

Proximate/antioxidant analyses	*P. emblica* (mean% ± SD)	*P. ginseng* (mean% ± SD)
Ash	2.28 ± 0.14	6.05 ± 0.73
Protein	2.2 ± 0.04	14.6 ± 1.88
Carbohydrate	80 ± 0.81	65.6 ± 2.39
Fiber	11.0 ± 1.47	4.83 ± 0.51
Moisture	5.6 ± 0.2	12.2 ± 0.48
Fat	0.14 ± 0.01	1.66 ± 0.34
Antioxidant composition (IC 50 μg/mL)	309.26 ± 3.08	13.88 ± 1.26

The proximate analysis of *P. ginseng* powder yielded results that are consistent with previous literature, where similar proximate values were observed in cream soup containing ginseng powder but the moisture levels differed significantly due to the difference between fresh ginseng root and dried ginseng powder samples (Kwon et al. 2022). The composition of ginseng exhibited ash 6.05%, protein 14.6%, carbohydrate 65.6%, fiber 4.83%, moisture 12.2%, and fat 1.66%. These values are consistent with past literature where, supplementation of red ginseng extract in milk was found to preserve stable protein and fat levels, indicating minimal impact of ginseng on basic nutritional composition (Jung et al. 2015). Such concordance enhances confidence in the present findings and highlights *P. ginseng* powder's potential as a nutritionally stable ingredient.

### 3.2 Antioxidant Analysis of *P. emblica* and *P. ginseng*

The antioxidant capacity of *P. emblica* through the DPPH radical scavenging method exhibited an IC50 (half maximal inhibitory concentration) value of 309.27 ± 3.09 μg/mL, reflecting a moderate level of antioxidant activity. The present findings align with prior research which demonstrated that the methanolic extract of *P. emblica* exhibited marked radical scavenging potential linked to its polyphenol and flavonoid content (Sharif et al. 2023). The observed discrepancies in IC50 values could be attributed to differences in extraction techniques, plant composition, or experimental design. Overall, these results reaffirm the bioactive constituents of *P. emblica* 's bioactive compounds in conferring antioxidant activity.

The antioxidant potential of *P. ginseng* was calculated using DPPH assay, which yielded 13.88 ± 1.26 μg/mL, reflecting strong antioxidant activity. This result is consistent with Wu and Wang (2008) where an arabino‐glucogalactan from *P. ginseng* root exhibited marked DPPH scavenging activity. Ginsenosides and polysaccharides are the primary contributors to the antioxidant strength of *P. ginseng* , owing to its free radical scavenging activity.

### 3.3 Hepatic Enzymes

Table 3 represents the serum levels of GGT, AST, ALT, and ALP in experimental groups.

**TABLE 3:** Effects of treatment on hepatic enzymes.

Treatment	GGT (U/L)	AST (U/L)	ALT (U/L)	ALP (U/L)
G0	16.71 ± 1.59^d^	126.12 ± 1.63^b^	47.62 ± 5.63^d^	175.12 ± 1.67^d^
G1	78.14 ± 1.91^a^	529.92 ± 51.83^a^	179.82 ± 13.59^a^	260.2 ± 1.35^a^
G2	68.64 ± 1.44^b^	131.70 ± 2.36^b^	70.07 ± 8.15^c^	206.65 ± 6.50^b^
G3	67.33 ± 2.04^b^	134.97 ± 0.99^b^	119.15 ± 9.06^b^	188.97 ± 5.27^c^
G4	56.54 ± 3.42^c^	124.72 ± 1.85^b^	57.10 ± 3.13^cd^	173.62 ± 3.78^d^

*Note:* Values with different letters in the same column (a–d) are significantly different (*p* < 0.05) from each other.

#### 3.3.1 GGT

In the present study, the liver fibrosis control group G1 showed significantly higher GGT levels compared to the normal control group G0, confirming induction of hepatic injury and fibrosis. G2 and G3 treatment groups led to a modest but statistically significant decrease in GGT levels between a range of 65–69 U/L approximately, as *P. emblica* is rich in vitamin C and polyphenols possessing strong activity against ROS. Whereas, ginseng in G3 enhanced the activity of antioxidant enzymes, mitigated oxidative stress, and recovered GGT levels. The combination treatment group G4 showed a notable decline in GGT levels due to the suppression of mitochondrial damage and inflammatory signals that are majorly involved in activation of HSCs, the key component in fibrosis progression (Gong et al. 2022). While the value of G4 is higher than the normal control G0, it is still statistically lower than the diseased group G1, demonstrating the combined efficacy of both medicinal plants against liver fibrosis. All treatment groups (G2, G3, and G4) led to a significant reduction in GGT levels, with G4 being the most effective among all treatments. All groups showed significant results (*p* < 0.05).

Elevated serum GGT levels as seen in G1 are strongly correlated with hepatic steatosis, inflammation, and oxidative stress in NAFLD, and prolonged increase are indicative of advanced fibrosis, reinforcing its role as a biomarker of hepatic injury and disease severity (Chen et al. 2021; Ha et al. 2022). Another study showed similar results where rabbits treated with APAP showed significantly high levels of GGT indicating hepatobiliary damage in response to oxidative stress. GGT serves as an important biomarker of liver dysfunction and is often upregulated in response to GSH depletion (Khaled and Saad 2025). Similarly in another study, that evaluated the effects of methanolic extract of dragon fruit against APAP‐induced hepatotoxicity, GGT was high in APAP group indicating liver damage (Parmar 2019). Treatment groups G2 and G3 produced notable reduction in GGT levels as observed in past literature Hafez et al. (2017) who proved the anti‐fibrotic effect of ginseng by inhibiting transfer growth factor‐beta 1/Smad pathway which is a pro‐fibrotic pathway; however these parameters were not investigated in the present study. Another study where fermented ginseng was assessed for its positive effects on liver function showed that it lowered GGT levels significantly. This effect is mainly attributed to its bioactive ginsenosides, which enhance antioxidant defense, reduce lipid peroxidation and inhibit inflammation pathways (Jung et al. 2020). Similarly, a study performed by Mishra (2022) concluded that aqueous extract of *P. emblica* effectively reduced the GGT levels and brought them closer to the control group. Whereas, the combination treatment group G4 demonstrated a significant decrease in GGT values. These results indicate partial hepatoprotection by individual treatments and an additive effect of combination therapy in mitigating APAP‐induced fibrosis, as evident by reduced GGT.

#### 3.3.2 AST

Significantly elevated values of AST were observed in the APAP group (G1), which indicates substantial liver damage that caused liver fibrosis. Whereas, the normal group (G0) maintained the baseline levels of AST within physiological ranges. When compared with the combination treatment group G4, that obtained the value closest to the normal group G0 and demonstrated a remarkable difference from the liver fibrosis group AST numerical value. Furthermore, G2 and G3 also attenuated liver fibrosis and gave values lower than G1. But G4 exhibited near‐complete reversal of APAP‐induced liver fibrosis. The AST levels differed significantly (*p* < 0.05) among all experimental groups. The APAP group (G1) exhibited significantly elevated AST, whereas treatment with G2, G3, and G4 led to a substantial decrease in AST levels, which aligned with the values of the normal control (G0) group. This indicated that the interventions effectively mitigated liver fibrosis.

The APAP group G1 demonstrated high levels of AST, confirming hepatocellular injury and impaired hepatic function, consistent with the well‐established mechanism of APAP toxicity involving NAPQI‐ mediated oxidative stress and hepatocyte necrosis (Wu et al. 2019). In contrast, the normal control group maintained a stable range within the normal physiological range, exhibiting intact hepatic function. *P. emblica* , *P. ginseng* and their combination treatment significantly reversed drug induced alterations, restoring AST levels. All treatment groups indicated comparable efficacy in treatment of liver fibrosis and restoring AST levels, but the G4 group showed the values closest to that of G0. The observations of G2 aligned well with the previous literature that showed its protective effects against lead‐induced toxicity, through attenuating oxidative stress (Paul et al. 2022). Similarly, *P. ginseng* treatment reduced AST levels which may be associated with its previously reported antioxidant mechanism which includes the activation of nuclear factor erythroid 2‐ related factor 2/heme oxygenase‐1 (Nrf2/HO‐1) pathway, which restores GSH and antioxidant enzymes while reducing lipid peroxidation, attributed to its antioxidant compounds; however, this mechanism was not directly evaluated in the present study (Wang et al. 2018). The combination treatment results aligned well with previous reports suggesting that *P. emblica* and *P. ginseng* enhance therapeutic efficacy through their bioactive components quercetin, gallic acid, ellagic acid and ginsenosides Rg1, Rg3, Rg5, Rg5/Rc which inhibit apoptosis and enhance antioxidant activity respectively (Rameshrad et al. 2024). Overall, the findings substantiate the role of both *P. emblica* and *P. ginseng* , individually and combined in mitigating APAP‐induced liver fibrosis.

#### 3.3.3 ALT

ALT levels showed significant variations among the experimental groups. The APAP group (G1) exhibited the highest levels of ALT showing liver fibrosis was induced, whereas the control group (G0) exhibited the lowest values indicating normal liver function. *P. emblica* group (G2) significantly reduced the level of ALT, demonstrating notable hepatoprotective efficacy; in contrast, *P. ginseng* (G3) presented a moderate reduction in ALT levels owing to the antioxidant and anti‐fibrotic properties of both, whereas the combination treatment group (G4) showed the maximum decline in ALT levels which was close to the control group values showing the strong radical scavenging and immunomodulatory activities of *P. emblica* and *P. ginseng*. Statistically significant differences (*p* < 0.001) among all groups confirmed variable degrees of effectiveness in mitigating liver damage.

When we compare these findings to the previous research, the normal control group G0 remained in the standard physiological range, confirming the intact hepatic function (Chaphalkar et al. 2017). G1 exhibited elevated levels as seen in previous research which showed that ALT, AST levels peaked after APAP administration (Okike et al. 2025). Treatment with *P. emblica* (G2) findings were also consistent with scientific literature which demonstrated the hepatoprotective and antioxidant effects against copper‐induced toxicity (Zulfiqar et al. 2023). The ameliorative effect of *P. ginseng* against increased ALT levels in G3 may be attributed to its antioxidant activity due to bioactive components such as ginsenosides, polyacetylenes, flavonoids, and phenolics (Ghamry et al. 2022). The combination treatment G4 result was in accordance with scientific research demonstrating the hepatoprotective effects of *P. emblica* with polyherbal formulations (Panchabhai et al. 2008).

#### 3.3.4 ALP

Approximately 10% of patients with NAFLD and nonalcoholic steatohepatitis (NASH) show elevated levels of serum ALP, and nearly one third of these patients are diagnosed with advanced fibrosis, highlighting ALP as a biomarker in disease progression (Ali et al. 2021). The positive control group (G1) showed highest levels of ALP, indicating significant liver fibrosis due to oxidative stress and hepatocyte damage induced by overdose of APAP. The normal control group (G0) maintained ALP baseline levels reflecting normal liver function. Administration of *P. emblica* in the G2 group, ALP levels dropped likely due to the high content of ascorbic acid and gallic acid which help in scavenging free radicals and reducing oxidative stress. Treatment with *P. ginseng* lowered ALP owing to the presence of ginsenosides that suppress oxidative stress and lipid peroxidation. G4 showed the most pronounced results, which implies that the combined effect of both ethnomedicinal plants restored liver function. Comparatively, G4 achieved the best results among all treatment groups and proved to be slightly greater in terms of hepatoprotective effect. A statistically significant (*p* < 0.001) was observed among study groups, reflecting the extent of fibrosis and the efficacy of treatments against reduction of fibrotic progression, as evident by different superscript letters.

The APAP group G1 exhibited elevated levels of ALP, which confirms the presence of hepatic injury when compared to the normal group (G0). This is consistent with the findings of previous literature, where acute and chronic administration of APAP showed elevated levels of ALP marking the hepatotoxic and fibrogenic effect of the drug (Abdel‐Hamid et al. 2022). Another study where mice were injected with high and low doses of APAP, the high dose group (1 g/kg/day) showed high levels of hepatic enzymes (ALP, AST, ALT) (Merah 2025). Administration of *P. emblica* (G2) significantly attenuated ALP levels, demonstrating partial hepatoprotection according to previous reports which highlight the key antioxidant compounds such as gallic acid and ellagic acid in *P. emblica* and another study which demonstrated its effectiveness against copper‐induced hepatotoxicity (Chaphalkar et al. 2017; Zulfiqar et al. 2023). *P. ginseng* (G3) produced a greater protective effect, consistent with its reported antioxidant and anti‐inflammatory activities (Ghamry et al. 2022). Notably, the combination treatment (G4) yielded the most pronounced reduction (173.62 ± 3.78 U/L), suggesting combined hepatoprotective efficacy, corroborating earlier observations on the enhanced therapeutic potential of combined herbal intervention (Panchabhai et al. 2008).

### 3.4 Bilirubin and Protein Markers

The serum total bilirubin, direct bilirubin, and albumin levels in experimental groups are shown in Table 4.

**TABLE 4:** Effects of treatment on bilirubin and protein markers.

Treatment	Total bilirubin (mg/dL)	Direct bilirubin (mg/dL)	Albumin (g/dL)
G0	0.67 ± 0.12^d^	0.65 ± 0.18^c^	5.57 ± 0.43^a^
G1	13.42 ± 1.40^a^	7.25 ± 0.51^a^	2.39 ± 0.11^d^
G2	6.2 ± 0.48^b^	4.3 ± 0.39^b^	3.71 ± 0.19^c^
G3	2.65 ± 0.53^c^	0.36 ± 0.08^c^	3.78 ± 0.18^c^
G4	1.87 ± 0.10^cd^	0.43 ± 0.03^c^	4.73 ± 0.37^b^

*Note:* Values with different letters in the same column (a–d) are significantly different (*p* < 0.05) from each other.

#### 3.4.1 Total Bilirubin

The serum bilirubin levels across experimental groups show the extent of fibrosis and efficacy of therapeutic interventions. Significant difference (*p* < 0.05) among groups was observed, where highest total bilirubin levels were observed in APAP‐induced liver fibrosis group G1. Elevation in bilirubin level was observed due to APAP toxicity and production of NAPQI, which demonstrated hepatotoxicity ultimately due to oxidative stress and hepatic necrosis. This further activated inflammatory pathways and hepatic stellate cells which led to fibrosis. The normal control group G0 possessed lowest bilirubin levels which indicated a normal liver function and efficient bilirubin clearance. G2 showed moderately reduced levels for bilirubin which limited the oxidative stress and reduced NAPQI by excreting it from the body. Whereas G3 produced a larger drop than G2, owing to ginsenosides present in ginseng that exert antioxidant effect, stimulate hepatic cell repair and restore bile flow more accurately than *P. emblica* . The combination treatment group G4 showed the best outcomes when compared to G2 but did not show a significant difference when compared to G3. This revealed that although both *P. emblica* and *P. ginseng* are hepatoprotective, ginseng provides protection beyond amla alone in respect to this parameter. G4 was not significantly different from *P. ginseng* which indicated the dominant effectiveness of ginseng within this framework.

Serum bilirubin contributes to cell protection and reduced risk of metabolic and cardiovascular diseases, as it exhibits antioxidant and anti‐inflammatory properties (Kwak et al. 2012). Although elevated bilirubin levels are recognized as liver dysfunction biomarker in ailments like NAFLD, they may have a dual function by influencing the pathophysiology of liver diseases and offering potential avenues for therapeutic intervention (Kamisako et al. 2000). Serum bilirubin analysis demonstrated significant differences among groups, reflecting both liver injury severity and therapeutic effects. The normal control (G0) maintained the lowest bilirubin levels, indicating normal hepatic function. APAP‐induced liver fibrosis group (G1) exhibited the highest levels of bilirubin, demonstrating liver dysfunction in accordance with previous study which clearly states that bilirubin accumulation is a critical clinical indicator of liver necrosis severity and indicative of the functional capacity of hepatocytes in APAP‐induced liver damage (Parmar 2019). Treatment with *P. emblica* (G2) moderately reduced bilirubin, which may be attributed to its hepatoprotective effect. Yin et al. (2021) demonstrated that aqueous extract of *P. emblica* ameliorated CCl_4_‐induced liver fibrosis by reducing oxidative stress, suppression of inflammatory cytokines, and downregulating profibrotic markers. Nawaz et al. (2022) reported that *P. emblica* leaf extract enhanced bilirubin clearance in phenylhydrazine‐induced hyperbilirubinemia rabbit model, in a dose dependent manner, supporting the bilirubin restoration effect observed in current study. *P. ginseng* group (G3) produced a greater reduction, the results thus align with the research performed by Yao et al. (2020), where *P. ginseng* hepatoprotective effects on bilirubin were not directly reported but the research significantly highlighted its effects against APAP‐induced liver toxicity by improving lipid peroxidation and oxidative stress, lowering the activity of pro‐inflammatory cytokines and cell death. *P. ginseng* restored elevated levels of bilirubin in thioacetamide (TAA)‐induced liver injury which is attributed to ginsenosides that significantly enhanced total antioxidant capacity (TAC), reduced oxidative stress. Although these parameters were not evaluated in the present research, past literature highlights the capability of ginseng in suppression of inflammatory markers including TNF‐α (tumor necrosis factor alpha), AGEs (advanced glycation end products), and NF‐_K_B (nuclear factor kappa‐light‐chain‐enhancer of activated B cells) signaling (Mostafa et al. 2021). The combination treatment group G4 achieved the most significant results, supporting combined benefits, consistent with Parveen et al. (2021) where therapeutic effects of multi‐herbal formulation of *P. emblica* , *Tinospora cordifolia*, and *Piper nigrum* against immunosuppressed mice were proved.

#### 3.4.2 Direct Bilirubin

When we compare bilirubin levels across treatment groups, the difference in hepatoprotective potential is evident. A significant difference is shown among groups (*p* < 0.05), G3 having the stronger impact on direct bilirubin levels. APAP group (G1) displayed the highest levels of serum bilirubin levels (7.25 ± 0.51) which confirms liver dysfunction. *P. emblica* (G2) group significantly lowered bilirubin levels relative to G1 (i.e., 4.3 ± 0.39). Whereas the bilirubin levels of G2 group remained elevated as compared to normal control group, indicating incomplete recovery. G3 (0.36 ± 0.08) and G4 (0.43 ± 0.03) possessed bilirubin levels comparable to G0, reflecting nearly complete restoration of liver function. Further, G3 showed that *P. ginseng* has stronger effect on direct bilirubin levels than *P. emblica* . Similarly, G4 showed a remarkably lower level of bilirubin than G2 identifying the significant potential of combined treatment groups when compared to *P. emblica* . Whereas, when G4 was compared to G3 it showed nearly no significant difference between both groups, indicating that the combined treatment does not exceed the efficacy of *P. ginseng* alone.

G1 group showed results according to the previous research where paracetamol administration caused elevation in bilirubin levels (Islam et al. 2021). The results of G2 align with the past research literature, which showed that the aqueous extract of *P. emblica* attenuated hepatic steatosis and fibrosis in high fat‐diet induced NAFLD through antioxidant activity and regulation of lipid metabolism (Huang et al. 2017). The present findings of *P. ginseng* group (G3) are consistent with previous literature, which illustrated the high contents of Rg1 and Rb1 ginsenosides in ginseng, where Rg1 possesses strong antioxidant and anti‐inflammatory properties and Rb1 contains anti‐fibrotic effects and inhibits liver stellate cells activation (Lee et al. 2015). The combination treatment results are also in accordance with Naz and Abbas (2016) suggesting that *P. emblica* with silymarin against cisplatin induced hepatic toxicity in combination therapy exerts therapeutic effects.

#### 3.4.3 Albumin

Albumin is a major protein synthesized by the liver. It serves as an important biomarker of liver function, and its reduction indicates impaired protein synthesis and fibrosis progression (Jagdish et al. 2021). In the current research, serum albumin levels were markedly reduced in the APAP group (G1), confirming hepatic synthetic dysfunction, whereas the normal control (G0) maintained values within the physiological range. Treatment with *P. emblica* (G2: 3.71 ± 0.19 g/dL) and *P. ginseng* (G3) moderately improved albumin concentration near G0 values, reflecting their hepatoprotective effects. Notably, the combination therapy (G4) achieved a more pronounced recovery, approaching normal values of the G0 group, suggesting combined benefits of the two herbal agents. All the groups indicated a significant result (*p* < 0.05), with G4 showing the most pronounced effect on albumin levels.

These results are in accordance with earlier reports as there was an improvement in albumin levels observed with *P. emblica* and *P. ginseng* , and their combined treatment, owing to the hepatoprotective effects of both ingredients. The best outcomes were demonstrated by G4 group due to the combined hepatoprotective potency of ingredients. These results align with past research, which showed that TAA and CCl_4_ treated rats exhibited an impaired level of total protein, albumin and globulin indicating severe hepatic injury. Rats were treated with apigenin which ameliorated the reduction of albumin. The improvement resembled G2 group. Another research demonstrated a decline in albumin due to APAP which resembled to the G1 group of present study and treatment comprised of rosmarinic acid that attenuated albumin reduction, whereas greater improvement was observed in G3 and G4 treatment groups (Alamri 2024; Elangovan et al. 2018; Hasanein and Sharifi 2017).

### 3.5 Oxidative Stress and Hematological Markers

Table 5 demonstrates the measured values of GSH, white blood cells (WBCs), platelets, and red blood cells (RBCs).

**TABLE 5:** Effect of treatment on oxidative stress and hematological parameters.

Treatment	GSH (μmol/g)	WBCs (×10^3^/mm^3^)	Platelets (%)	RBCs (× 10^6^μL)
G0	6.67 ± 0.63^a^	10.92 ± 0.81^b^	0.2175 ± 0.014^b^	9.16 ± 0.67^a^
G1	4.51 ± 0.39^b^	6.45 ± 0.26^c^	0.3900 ± 0.071^a^	6.51 ± 0.45^b^
G2	5.29 ± 0.41^bc^	11.21 ± 0.11^b^	0.2325 ± 0.034^b^	6.29 ± 1.41^b^
G3	4.44 ± 0.40^c^	10.94 ± 0.42^b^	0.2000 ± 0.018^bc^	5.04 ± 1.07^b^
G4	4.68 ± 0.28^c^	15.29 ± 1.25^a^	0.1395 ± 0.02^c^	4.85 ± 1.27^b^

*Note:* Values with different letters in the same column (a–d) are significantly different (*p* < 0.05) from each other.

#### 3.5.1 GSH

Liver fibrosis is closely linked with impaired GSH homeostasis, where reduced GSH promotes oxidative stress and fibrogenesis, making its maintenance essential for hepatoprotection (Li et al. 2024). The reduction of GSH levels is directly related to its consumption during detoxification of APAP metabolite NAPQI (Bhattacharyya et al. 2013). Pivotal studies demonstrate that GSH depletion occurred rapidly after APAP administration in a time and dose dependent manner, with more than 80% of total hepatic GSH depleted within 2 h following a highly hepatotoxic dose of 400 mg/kg APAP. GSH depletion is a crucial step in the pathophysiology of APAP‐induced liver toxicity, as evident by the important finding that it occurs prior to the covalent binding of the reactive metabolite to cellular proteins and that this covalent binding precedes the initiation of hepatocellular injury (Jollow et al. 1973; McGill and Hinson 2020; Mitchell, Jollow, Potter, Gillette, and Brodie 1973). Studies have reported that overdose of APAP reduced GSH levels to 60% than control group after 6 h of dosing, which makes it the key antioxidant in early detection of liver dysfunction caused by APAP overdose (Powell et al. 2007). Although GSH is a well‐established mechanistic marker of APAP‐induced oxidative stress, inclusion of additional oxidative stress biomarkers (e.g., superoxide dismutase, catalase, malondialdehyde) would provide a broader characterization of the antioxidant response and should be considered in future studies.

In this study, the normal control group (G0) showed the highest GSH levels, while the APAP group (G1) exhibited significant depletion, confirming oxidative stress. *P. emblica* (G2) partially restored GSH, indicating moderate antioxidant potential; on the other hand, *P. ginseng* (G3) and the combination group (G4) showed comparatively lower levels, suggesting limited efficacy under current conditions. Although G2 achieved partial recovery, G3 and G4 did not differ significantly from G1, implying the need for dose or duration optimization. Overall, APAP caused elevated levels of biomarkers such as ALT, ALP, and GSH, while both *P. emblica* and *P. ginseng* improved these biomarkers by elevating oxidative stress and modulating hepatic injury indicators by anti‐inflammatory pathways. Overall, all the groups indicated a significant (*p* < 0.05) difference, with G2 showing the strongest antioxidant potential, owing to the presence of gallic acid, ellagic acid, and ascorbic acid. The outcome of *Moringa oleifera* research closely resembles the findings of the present study. In both models, NaNO3 (Sodium nitrate) or APAP caused liver fibrosis, whereas plant‐treated groups such as *M. oleifera* and *P. emblica* reversed the damage caused by the drug (Fouad et al. 2025). Notably, the G2 group with *P. emblica* showed the greatest hepatoprotective effects, owing to the superior antioxidant activity (Mochahary 2024). Another study demonstrated that *P. emblica* improved GSH levels in patients with diabetes mellitus type 2, owing to its bioactive components such as tannins, emblicanin A, and emblicanin B (Usharani et al. 2013). However, *P. ginseng* showed limited recovery, which may be attributed to differences in its preparation, dose, duration of treatment, and experimental conditions. Moreover, the severity of APAP‐induced oxidative stress may have exceeded the capacity of ginseng to replenish the glutathione stores. The failure of the combination treatment in the recovery of oxidative stress highlights the complex nature of herb‐to‐herb interactions. Previous literature states that herbal treatments are often expected to show strong combined activity, but their efficacy is often influenced by the mechanism of action, individual proportion of herbal components, and requirement for common cellular resources, making it difficult to predict the interaction of different herbs (Prakash et al. 2009). Another study where ginseng and licorice root extract combinations had antagonistic effects on cell proliferation and enhanced the survival of hepatocarcinoma cells (Hep‐G2), whereas the individual treatment reduced Hep‐G2 viability. This indicates that the biological response of a herbal combination cannot be predicted based on the effects of its individual components (Popovich et al. 2011).

#### 3.5.2 White Blood Cells (WBCs)

WBC was used as a general indicator of inflammation associated with APAP‐induced hepatic injury. Although differential leukocyte count (DLC) could provide additional information regarding immune cell populations, the present study mainly focused on biochemical, hematological, oxidative stress, and histopathological assessments. WBCs are special neutrophils that significantly contribute to liver fibrogenesis by promoting inflammation. Elevated levels of the neutrophil to lymphocyte ratio (NLR) have been shown to correlate with advanced liver fibrosis in NAFLD, supporting its role as a noninvasive biomarker for disease evaluation (Wang et al. 2024).

However, in the current study suppressed WBC levels were found in only the APAP group (G1), which can be explained by bone marrow suppression and hematological toxicity. The APAP group (G1: 6.45 ± 0.26) demonstrated a significant reduction in WBCs level when compared to the normal control group (G0: 10.92 ± 0.81). Conversely, treatment with *P. emblica* (G2: 11.21 ± 0.11) and *P. ginseng* (G3: 10.94 ± 0.42) restored the WBCs level and brought them closer to the normal values, reflecting that compounds in *P. emblica* such as gallic acid, ellagic acid, and ascorbic acid have strong free radical scavenging activity whereas ginseng possesses immunomodulatory activity and protects bone marrow from oxidative stress. The combination treatment (G4: 15.29 ± 1.25) showed the most efficient results out of all the treatment groups. These findings align with previous research highlighting the immunomodulatory capacity of both *P. emblica* and *P. ginseng* in supporting recovery from hepatic injury (Akter et al. 2020; Gotlieb et al. 2020; Kapoor et al. 2019). The results revealed a significant difference (*p* < 0.05) in WBCs level among all groups.

The reduction in WBC count in the APAP group (G1) is consistent with earlier research where APAP administration reportedly compromised immune activity, caused by bone marrow suppression which ultimately led to reduced WBCs that was restored by thyme extract (Mokhtari et al. 2023; Raghavendran et al. 2012). The restoration of WBC levels in *P. emblica* (G2) and *P. ginseng* (G3) group supports evidence that these herbal treatments exert immunomodulatory and anti‐fibrotic effects, promoting leukocyte recovery and liver regeneration (Pryuttma and Khan 2020). The results of the G4 group align well with previous literature where it was reported that herbal therapy resulted in remarkably improved hematological parameters in a liver fibrosis experimental model (Zheng et al. 2019).

#### 3.5.3 Platelets

A significant difference (*p* < 0.05) was observed among groups. Platelet levels were significantly high in the APAP group (G1: 0.3900 ± 0.071) when compared to the normal control (G0: 0.2175 ± 0.014), reflecting reactive thrombocytosis associated with inflammation and fibrosis. An APAP overdose creates a violent rebalancing of the body's clotting and immune systems (Rajala et al. 2025). Treatment with *P. emblica* (G2: 0.2325 ± 0.034) and *P. ginseng* (G3: 0.2000 ± 0.018) effectively reduced platelet counts towards normal, demonstrating their immunomodulatory effectiveness. The combination treatments (G4: 0.1395 ± 0.02) had the lowest values of platelets, suggesting the combined effect owing to the antioxidative and anti‐inflammatory effects of both treatments.

The acute damage from NAPQI rapidly depletes GSH which results in centrilobular liver necrosis, causing inflammatory cytokines to flood the bloodstream. In response to sudden tissue injury, the body undergoes an acute‐phase reaction where bone marrow is forced to accelerate the production of platelets. This causes a prominent spike in platelet level as seen in G1 (Gosselin et al. 2014; Mahmoudi et al. 2015). The platelet restoration of *P. emblica* may be attributed to its rich phenolic and flavonoid content, particularly quercetin, which possesses antioxidant and hepatoprotective properties, as reported in previous studies where its extract accelerated platelet recovery in a thrombocytopenia rat model (Chawansuntati et al. 2024; Pratima et al. 2025). Furthermore, ginsenosides in *P. ginseng* may regulate platelet level through modulation of glycoprotein VI, which is a receptor on the platelet surface that causes its activation (Akram et al. 2025). G4 platelet count was below G0 group, which may indicate anti‐fibrotic activity and potentially an increase towards platelet consumption for liver repair. These findings are consistent with previous reports showing that natural antioxidants and herbal formulations help normalize platelet counts and attenuate hepatic inflammation and fibrosis. A research study showed that combined treatment of *Ficus capensis* and *Mentha spicata* showed anti‐inflammatory and platelet modulation activity (Asadu et al. 2026). The results align with the present study. The treatments showed improvement in platelet level where combination treatment proved to be most effective.

#### 3.5.4 Red Blood Cells (RBCs)

RBC levels differed significantly among all groups (*p* < 0.05); however, treatment groups were not significantly different from each other as shown by the subscript letters. RBCs level across the treatment groups demonstrates noticeable changes due to the APAP‐induced liver fibrosis and subsequent interventions. Highest RBC level was observed in normal group (G0) that aligns within the physiological ranges. G1 exhibited a significant reduction indicating toxicity effects likely due to oxidative stress, impaired erythropoiesis, and liver dysfunction (Suhail and Ahmad 1995). APAP is widely known to cause hematological disturbances in rodent models. When compared to G0, *P. emblica* group (G2) showed nonsignificant results. The G3 group possessed decreased RBC levels when compared to G1 group; this suggested a limited hepatoprotective effect on erythropoiesis under APAP‐induced fibrosis. lowest RBC levels, indicating high variability in the group. The combined treatment G4 presented the lowest value in comparison to all other treatment groups.

Although G2, G3, and G4 exerted significantly improved most parameters, RBC levels did not recover and were lowest in combination group G4. The lack of improvement in RBC levels despite improvements in other parameters may be due to inherent biological variability among animals, which could have masked subtle treatment effects.

Treatment with *P. emblica* and *P. ginseng* individually and in combination resulted in further reduction in RBC counts; the combination group exhibited the greatest decrease despite showing pronounced results in biochemical and histopathological indicators of liver fibrosis. This discrepancy suggests that the hepatological response to these interventions may differ from their hepatoprotective effects. As the present study was primarily designed to evaluate liver fibrosis rather than hematological mechanisms, the reason for the observed reduction in RBC counts remains unclear and requires further investigation.

The RBC counts across the experimental groups revealed significant alterations associated with APAP‐induced liver fibrosis and subsequent treatments. The normal control group (G0: 9.16 ± 0.67) exhibited the highest RBC values, consistent with a normal hematological profile (Prananda et al. 2023). In contrast, the APAP group (G1: 6.51 ± 0.45) demonstrated a pronounced reduction, reflecting blood toxicity likely attributable to impaired hepatic function, oxidative stress, or disrupted erythropoiesis (O'Brien et al. 2000). Comparable reductions in RBC levels following APAP exposure have been documented in previous literature Liang et al. (2024), reported that APAP toxicity can cause hematological disturbances, and changes that ginseng is reported to counteract, due to the ginsenosides (Rb1, Rb2, Rg1, Rg2, Rc, Rd). *P. ginseng* treated group (G3: 5.04 ± 1.07) showed a greater decline than G2 in RBC counts, whereas in previous researches ginseng proved to exert hematopoietic protective effect by supporting hematopoietic stem cell survival and reducing apoptosis (Liang et al. 2024). This may suggest that the hematopoietic protective effects suggested in literature may not translate under APAP‐induced liver fibrosis model or at the administrated dose or duration. Administration of *P. emblica* (G2: 6.29 ± 1.41) produced only a modest and statistically insignificant improvement. Whereas, *P. emblica* has been reported to exert antioxidant and improved hematic effects by study performed by (Prananda et al. 2023). The combination treatment (G4: 4.85 ± 1.27) exhibited the lowest RBC count, indicating no improvement, reduced bioavailability, or compounded metabolic stress (Zahiruddin et al. 2022). These results collectively indicate that the potential of both *P. emblica* and *P. ginseng* in restoring RBC level under severe hepatotoxic conditions appeared limited warranting further investigation into optimized formulations, dosages, or adjunct therapeutic strategies.

### 3.6 Histopathological Examination

**FIGURE 1:**
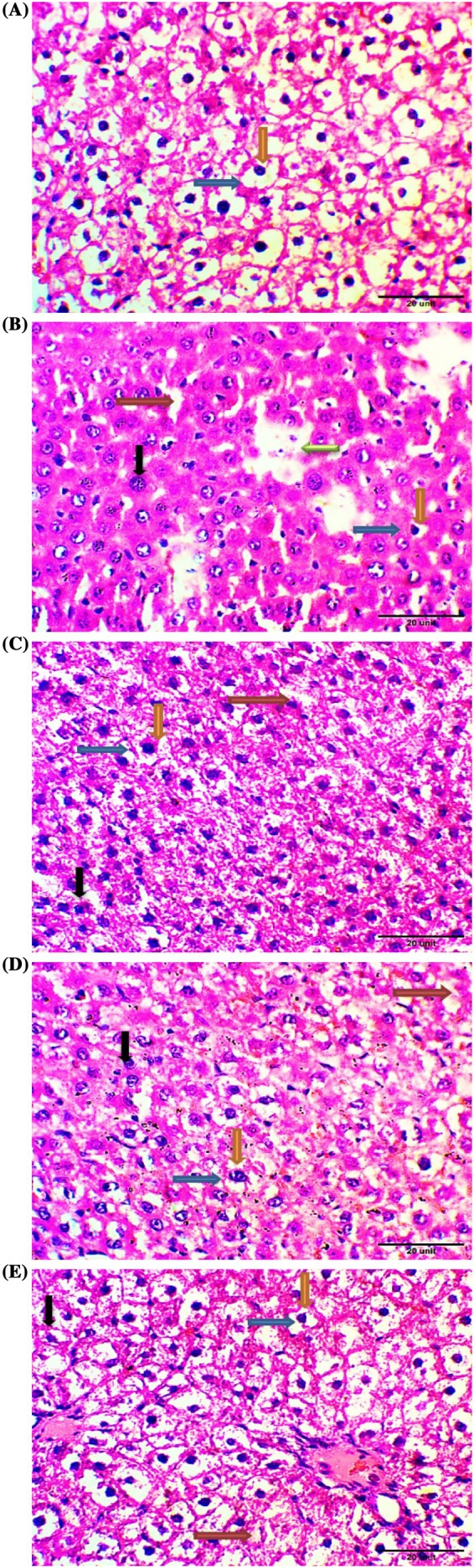
(A) Central nucleus, intact sinusoids and normal morphology with radiating hepatic chords from central vein is preserved in control group. (B) APAP‐treated animals exhibit widened sinusoids, steatosis, displaced nucleus, fibrosis, and degeneration. (C) *P. emblica* treated group G2 demonstrate mild restoration in hepatic architecture, with few pyknotic nuclei and reduced steatosis. (D) *P. ginseng* treated group G3 showed mild improvement, minor degenerative changes, still sinusoidal congestion and cellular infiltration (inflammation score 2). (E) G4 showed clear sinusoidal spaces, central nuclei, minimal suggested fibrosis.

Histopathological analysis of the liver in all experimental groups is illustrated in Figure 1. The electron microscope was used to capture the snaps of liver tissue at the magnification power of 400×. Hepatic lobules of control rats were intact, and the hepatocytes were arranged trimly without visible abnormalities. However, extensive diffuse necrosis of hepatocytes, steatosis, inflammatory infiltration, sinusoid congestion, and balloon‐like changes were detected in hepatic tissue of model rats, which demonstrated the liver fibrosis model was successfully established. Hematoxylin–eosin (H&E) staining was used for the initial histopathological observation of hepatic injury and fibrosis. Figure 1A shows that in G0 normal architecture of the liver tissue with hepatic cords radiating from the central vein, sinusoids were intact, no infiltration (inflammation score 0), absence of steatosis (score 0), and hepatocytes' nucleus was placed centrally without any abnormality. Figure 1B demonstrates that in G1 there were deviation and distortion from the normal histological pattern with widened sinusoids, extensive steatosis (score 3), inflammatory infiltration (inflammation score 3), displaced nucleus, and degenerative changes were seen in the hepatocytes suggesting the fibrosis‐like changes. In Figure 1C G2 treated with *P. emblica* provided moderate protection to the liver tissue as compared to the G1 group as hepatocytes are partially restored with few pyknotic nuclei and reduced steatosis (score 2). In Figure 1D G3 treated with *P. ginseng* showed mild improvement, mild degenerative changes, still sinusoidal congestion and cellular infiltration (inflammation score 2) than the *P. emblica*. G4 demonstrated marked improvement in liver histology, clear sinusoidal spaces, minimal suggested fibrosis‐related condition, hepatocytes with clear nuclei, reduced infiltration (inflammation score 1) and no steatosis (score 0). The present study demonstrated several histopathological changes that are suggestive of fibrosis, such as inflammatory infiltration, displaced nucleus, steatosis, and degenerative changes. Red arrows show sinusoidal space, orange arrows show the nucleus, blue arrows represent the hepatocytes, black arrows depict the infiltration of the inflammatory cells while green arrows show the vacuolization in between the hepatocytes (Enciso et al. 2022; Yin et al. 2021).

## 4 Conclusion

The present study demonstrated that supplementation with *P. emblica* and *P. ginseng* , either individually or in combination, exerted significant hepatoprotective effects against APAP‐induced liver fibrosis in a rat model. These treatments effectively improved biochemical, hematological, and histopathological parameters and brought them near to normal values. They showed significant efficiency in mitigating fibrosis progression. Notably, the combined treatment group G4 showed the greatest efficacy, closely resembling normal physiological values in most of the parameters, suggesting a potential combined effect. The combination of *P. emblica* and *P. ginseng* was most effective due to complementary bioactive compounds such as vitamin C, ellagic acid, and gallic acid from amla, together with ginsenosides and polysaccharides from ginseng, which enhanced hepatoprotective and antifibrotic effects, leading to improved liver enzyme profiles and restoration of normal hepatic histological architecture in the APAP‐induced fibrosis model. However, it was observed that *P. emblica* was more effective in restoring GSH levels, showing strong antioxidant activity in comparison to *P. ginseng* and their combination treatment, emphasizing the complex nature of herb‐to‐herb interactions. These findings provide strong experimental evidence supporting the therapeutic potential of these natural compounds in alleviating drug‐induced hepatic injury and highlight their promise as complementary interventions for managing liver fibrosis.

## 5 Future Perspective

The present study demonstrates the hepatoprotective and antifibrotic potential of *P. emblica* and *P. ginseng* against APAP‐induced liver fibrosis. Future studies should focus on examining important fibrogenic pathways, such as TGF‐β1 (tumor growth factor beta‐1), α‐SMA (alpha smooth muscle actin), collagen I/III, Nrf2 (nuclear factor erythroid 2‐related factor 2), and NF‐_K_B in order to validate the underlying molecular mechanism. A more thorough assessment of the treatment benefits would also be possible by evaluating fibrosis specific biomarkers such as hydroxyproline, matrix metalloproteinases (MMPs), and tissue inhibitors of metalloproteinases (TIMPs), in addition to other oxidative stress markers. *P. emblica* and *P. ginseng* powders should be phytochemically characterized and standardized in order to determine and measure their bioactive components, thereby improving reproducibility and facilitating comparison across studies. Future dose response studies should also determine the optimal therapeutic doses, treatment duration, and combination ratios of these powders. As previous studies have predominantly investigated plant extracts in liver disease models, further research focusing on the dose‐ and time‐dependent effects of powders in APAP ‐induced liver fibrosis would help bridge this knowledge gap. Finally, additional preclinical investigations, followed by rigorously designed clinical studies, are required to evaluate the safety, efficacy, and clinical translation of these botanical interventions for liver fibrosis.

## Author Contributions

**Fiza Javed:** conceptualization, data curation, formal analysis, writing – original draft. **Nourah Harbi Almutairi:** conceptualization, data curation, writing – original draft. **Wardha Tariq:** data curation, writing – review and editing. **Mah Noor:** writing – review and editing. **Shiza Javed:** writing – review and editing. **Usman Haider:** data curation, writing – review and editing. **Ghalia Shamlan:** writing – review and editing. **Gholamreza Abdi:** supervision, writing – review and editing. **Rana Muhammad Aadil:** conceptualization, data curation, funding acquisition, resources, writing – review and editing, supervision.

## Ethics Statement

The animal study was approved by the Institutional Biosafety and Bioethics Committee (D#2272/ORIC) UAF, Pakistan. The study was conducted in accordance with the local legislation and institutional requirements. All procedures in the study were conducted in compliance with the ARRIVE 2.0 guidelines to ensure transparency and reproducibility.

## Conflicts of Interest

The authors declare no conflicts of interest.

## Data Availability

The data that support the findings of this study are available from the corresponding author upon reasonable request.
